# Cell Type-Specific Effects of *Fusarium* Mycotoxins on Primary Neurons and Astroglial Cells

**DOI:** 10.3390/toxins17080368

**Published:** 2025-07-25

**Authors:** Viktória Szentgyörgyi, Brigitta Tagscherer-Micska, Anikó Rátkai, Katalin Schlett, Norbert Bencsik, Krisztián Tárnok

**Affiliations:** Department of Physiology and Neurobiology, Institute of Biology, Eötvös Loránd University, H-1053 Budapest, Hungaryschlett.katalin@ttk.elte.hu (K.S.)

**Keywords:** *Fusarium* mycotoxin, FB1, deoxynivalenol, zearalenone, neuron, cell culture, astroglia, toxicity, multi-electrode array (MEA)

## Abstract

Fumonisin B1, deoxynivalenol (DON), and zearalenone (ZEA) are toxic secondary metabolites produced by *Fusarium* molds. These mycotoxins are common food and feed pollutants and represent a risk to human and animal health. Although the mycotoxins produced by this genus can cross the blood–brain barrier in many species, their effect on neuronal function remains unclear. We investigated the cell viability effects of these toxins on specified neural cell types, including mouse primary neuronal, astroglial, and mixed-cell cultures 24 or 48 h after mycotoxin administration. DON decreased cell viability in a dose-dependent manner, independent of the culture type. Fumonisin B1 was toxic in pure neuronal cultures only at high doses, but toxicity was attenuated in mixed and pure astroglial cultures. ZEA had significant effects on all culture types in 10 nM by increasing cell viability and network activity, as revealed by multi-electrode array measurements. Since ZEA is a mycoestrogen, we analyzed the effects of ZEA on the expression of estrogen receptor isotypes ERα and ERβ and the mitochondrial voltage-dependent anion channel via qRT-PCR. In neuronal and mixed cultures, ZEA administration decreased ERα expression, while in astroglial cultures, it induced the opposite effect. Thus, our results emphasize that *Fusarium* mycotoxins act in a cell-specific manner.

## 1. Introduction

The fumonisin B1 (FB1), deoxynivalenol (DON), and zearalenone (ZEA) mycotoxins are common pollutants in food and feed worldwide and represent risks to both human and animal health [1]. These toxic secondary metabolites are produced by molds of the *Fusarium genus*. Due to climate change, the habitat of these species has changed, and the new weather conditions support their mycotoxin production [2]. Mycotoxins are usually thermostable; therefore, they can be present in processed food and feed despite milling, extrusion, storage, or heating. Mycotoxins can induce a multitude of physiological effects. For example, FB1, the most prevalent fumonisin type, is a known cancer promoter [3] linked to human esophageal cancer in South Africa and China. It is hepato-, nephro-, and immunotoxic as well, caused by the mechanisms of oxidative stress, apoptosis, and the inhibition of sphingolipid biosynthesis [4]. Chronic exposure to DON leads to feed refusal, anorexia, immunotoxicity, gastroenteritis, and skin dermatitis based on its inhibitory action on protein biosynthesis and subsequent activation of mitogen-activated protein kinases [5,6]. DON can activate oxidative stress by elevating ROS (reactive oxygen species) production, triggering inflammatory and apoptotic pathways and compromising antioxidant mechanisms, with partial compensation by the Nrf2-HO-1 (nuclear factor erythroid 2-related factor 2-heme oxygenase-1) system, which can be overloaded at higher exposures [7,8]. ZEA also has immuno- and hepatotoxic effects and induces genotoxicity via DNA adduct formation [9]. Specifically, ZEA exposure has been shown to suppress Nrf2 expression and activity in various cell types, including jejunal epithelial and endometrial stromal cells [10,11]. Some studies also report that antioxidants (e.g., quercetagetin and kaempferol) can restore Nrf2 activity and mitigate ZEA-induced cytotoxicity, highlighting the central role of the Nrf2 pathway in cellular defense against ZEA cytotoxicity [12,13]. In addition, ZEA is a potent endocrine disruptor due to its binding capacity to both the ERα and ERβ estrogen receptors [14,15]. By misregulating the hormonal system, it can cause severe symptoms such as fertility disorders, reduced litter size and fetal weight, alterations in the reproductive tract of animals, or early thelarche and premature puberty in humans [9].

Besides the general physiological effects, exposure to these mycotoxins can cause neurological symptoms [1]. In the case of FB1, the best-known effect is the equine leukoencephalomalacia (ELEM) in horses [16]; however, FB1 has also been linked to neural tube defects in children [17]. Former studies have also shown that FB1 can pass through the blood–brain barrier (BBB) [16]. In vitro studies on neural cultures have demonstrated that FB1 causes diverse cellular effects by inhibiting axonal and dendritic growth [18,19] or altering myelin formation and oligodendrocyte maturation [20] by disrupting sphingolipid metabolism [21]. Based on data from different cell lines, FB1 can induce cell death via oxidative stress and/or apoptosis in a cell type-specific manner [22].

Due to its strong emetic properties, deoxynivalenol is also known as vomitoxin. It is able to exert a direct neuronal effect, since circulating DON or its metabolites can pass the BBB and accumulate in the brain [23,24,25]. DON has been reported to cause GABAergic neuronal activation [26] and stimulate the neuronal dopamine receptors [27,28]. In vivo, it has been reported to increase salivation in swine [29]. DON can cause neuroinflammation [30], modify feeding behavior leading to reduced food intake [31,32] and alter goal-directed, reward-driven behaviors in rats [26].

Limited data are available on the direct neurologic and/or neurotoxic effects of ZEA. In vivo, chronic ZEA administration decreased the brain protein levels of rats and affected the cellular oxidoreductive capacity by increasing superoxide dismutase (SOD) and decreasing glutathione (GSH) levels and glutathione peroxidase activity (GSH-Px), resulting in elevated levels of NO production and apoptosis [33]. The ZEA-induced formation of ROS and oxidative stress were also observed in human neuroblastoma cell lines [34,35,36]. On the other hand, ZEA exerted no effect on estrogen biosynthesis in glioblastoma cells [37]. In a few reports, the administration of ZEA or its derivatives led to positive outcomes on memory impairment and adult neurogenesis [38], or was neuroprotective by counterbalancing the effects of ovariectomy or β-amyloid (25–35)-induced oxidative/ER stress [39,40,41]. The ZEA-mediated regulation of the expression of ERβ or thyroid receptor α,β has also been recently reported on cerebellar granule cells [42,43]. Although these data reveal important neurological consequences of mycotoxin consumption, and purified mycotoxins are intensively tested on different cell lines to determine their primary effects on cell survival and metabolism [44,45], current knowledge on their cell type-specific effects within the brain is still limited.

Primary cultures enable us to investigate cells derived directly from the brain tissue. To unravel the neuron- or astroglia-specific processes upon mycotoxin treatment, pure neuronal as well as mixed cultures containing both neurons and astroglial cells and pure astroglia cultures were established from embryonic and postnatal mouse cortices or forebrains, respectively. In the present work, we compare the cell type-specific effects of FB1, DON, and ZEA to define the cellular targets of the different mycotoxins. We provide evidence that neurons and astrocytes can exert cell type- and concentration-dependent responses to mycotoxins and can counterbalance the mycotoxin-evoked effects on each other. In addition, we show that ZEA rapidly promotes spontaneous bursting activity and the synchronization of in vitro neuronal networks.

## 2. Results

### 2.1. The Presence of Astroglial Cells Attenuates the Cytotoxic Effect of FB1

**Figure 1:**
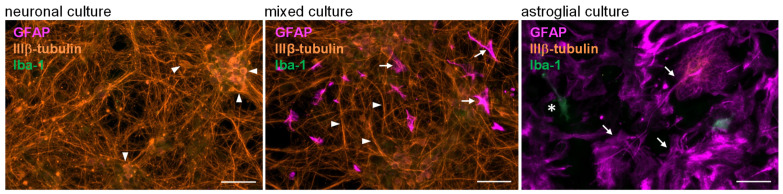
**Representative immunofluorescence images of three types of neural cultures applied for mycotoxin measurements.** Neurons (arrowheads), astroglial cells (arrows), and microglia (asterisk) were visualized using IIIβ-tubulin, glial fibrillary acidic protein (GFAP), and Iba-1 immunopositivity, respectively. Neuronal cultures (**left** image) were free of astroglial and microglial cells on DIV7, since mitosis was inhibited 24 h after plating by adding CAR. In mixed cultures (**middle** image), both neurons and astrocytes were detectable on DIV7. Astroglial cell cultures (**right** image) contained less than 2% microglia (asterisk), and they were free of neurons 24 h after seeding. Scale bars: 50 μm.

Neurons and astrocytes can exhibit diverse responses to mycotoxin administration. In order to determine and compare the cell type-specific effects of mycotoxins, three different types of primary cell cultures were created and used in our experiments containing (i) both neurons and astroglia (“mixed”), (ii) only neurons and no glial cells (“neuronal culture”), or (iii) neuron-free glial cells (“astroglial culture”). The cellular composition of cultures was verified via immunostaining using cell type-specific markers. Neurons, astroglia cells, and microglia were visualized using IIIβ-tubulin, glial fibrillary acidic protein (GFAP), and Iba-1 immunopositivity, respectively (Figure 1). In mixed cultures, both neurons and astrocytes were detectable. Neuronal cultures were free of astroglial and microglial cells, and while primary glia cultures contained a few microglial cells, they were mainly astrocyte-rich and neuron-free.

**Figure 2:**
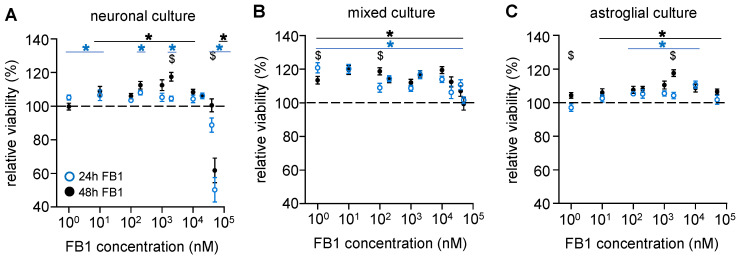
**Astroglial cells attenuate the cytotoxic effect of FB1 observed in neurons.** The effects of FB1 on the cell viability of mouse primary (**A**) neuronal, (**B**) mixed, and (**C**) astroglial cell cultures were determined after 24 (blue) or 48 h (black) treatments of 1, 10, 100, or 200 nM or 1, 2, 10, or 50 μM FB1 via MTT assay. Results are expressed in the percentage of control data, as mean ± s.e.m. “$” indicates significant differences between data obtained after 24 and 48 h treatment; * indicates significant differences compared to non-treated data (*: *p* < 0.05). *n* = 12–42.

To examine the cellular effects of FB1 mycotoxins, mixed and purified cultures were prepared in 96-well plates and treated for 24 or 48 h with increasing (1 nM–50 µM) concentrations of FB1. Cell viability was determined via the MTT assay. According to our data, FB1 significantly increased cell viability above a 10 nM concentration in cultures containing solely neurons or neurons and astroglia cells (Figure 2A,B). However, this effect was decreased above a 10 µM concentration, and in pure neuronal cultures, 50 µM of FB1 caused a drastic, 50% viability loss. Increasing the treatment from 24 to 48 h did not dramatically change the observed toxin-mediated effects.

In astroglial cultures, FB1 administration significantly increased cell viability above a 10 nM or 100 nM concentration in a 48 or 24 h treatment time, respectively (Figure 2C). The cytotoxic effects observed in pure neuronal cultures did not occur in glial nor in mixed cultures, so the presence of astroglial cells reduced the neuronal cytotoxicity of FB1.

### 2.2. Astroglial Cells Are More Sensitive than Neurons to the Cytotoxic Effects of DON

**Figure 3:**
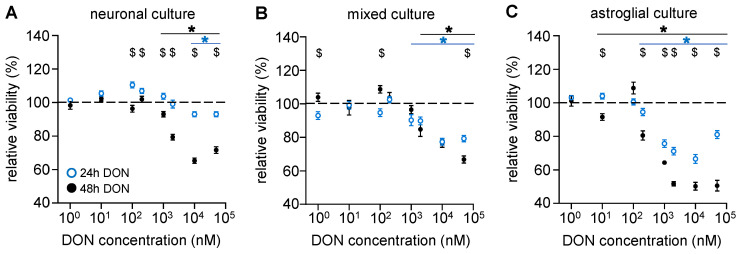
**Astroglial cells are the most sensitive to the cytotoxic effect of DON.** The effects of DON on the cell viability of mouse primary (**A**) neuronal, (**B**) mixed, and (**C**) astroglial cell cultures were determined after 24 (blue) or 48 h (black) treatments of 1, 10, 100, or 200 nM or 1, 2, 10, or 50 μM DON via MTT assay. Results are expressed in the percentage of control data, as mean ± s.e.m. “$” indicates significant differences between data obtained after 24 and 48 h treatment; * indicates significant differences compared to non-treated data (*: *p* < 0.05). *n* = 18–36.

In addition to FB1, *Fusarium* species also produce other mycotoxins, including deoxynivalenol (DON). To investigate the effects of DON on neural cells, cultures were treated similarly to in the FB1 experiments. Our results showed that in cultures containing either only neurons (Figure 3A) or both neurons and astrocytes (Figure 3B), DON treatment at concentrations below 1 µM was ineffective. In the concentration range of 1–50 µM, DON decreased cell viability in a concentration- and time-dependent manner. On the other hand, a more prominent, concentration-dependent decrease in viability was observed in astroglia cells after 24 h of DON treatment above 200 nM (Figure 3C). Toxicity was further elevated by 48 h of DON treatment and was evident from even lower concentrations (e.g., at 2 µM, 48 h treatment of DON resulted in an approx. 35% reduction in the viability of astroglia cells; Figure 3C). Notably, in mixed cultures, DON treatment reduced cell viability by only 20% at 2 µM (Figure 3B). Taken together, our data show that astroglial cells are the most sensitive to DON administration in our system.

### 2.3. ZEA Increases Cell Viability in All Investigated Culture Types Even at Low Concentrations and Alters the Expression of ERα in a Cell Type-Specific Manner

**Figure 4:**
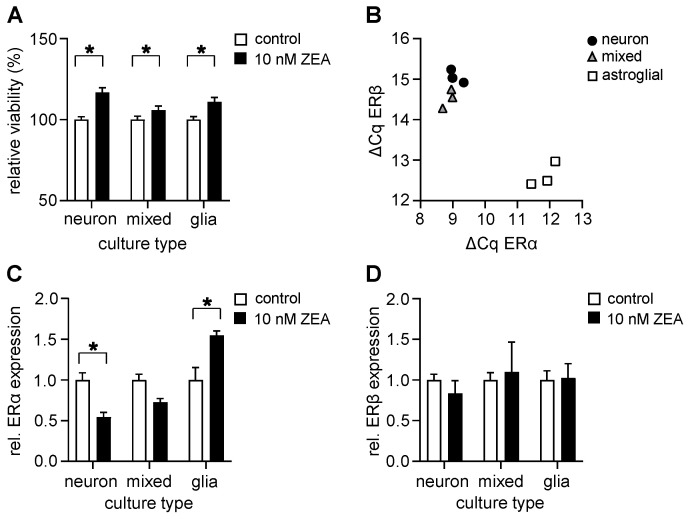
**Low ZEA concentration increases cell viability and alters the expression of ERα in a cell type-specific manner**. (**A**) Effects of 10 nM of ZEA on the cell viability of mouse primary neuronal, astroglial, and mixed-cell cultures after 48 h, as measured via MTT assay. Results are expressed in the percentage of control data, as mean ± s.e.m. *n* = 17–35. (**B**) ΔCq values of ERα and ERβ relative expression in the control, non-treated neuronal, astroglial, and mixed cultures, analyzed via qRT-PCR. Both estrogen receptor types are expressed in the different cell cultures. (**C**) qRT-PCR assay showed that in neuronal and mixed cultures, ZEA administration decreased, while in astroglial cultures, it increased ERα expression compared to the control cells. (**D**) ERβ expression, on the other hand, was not altered by ZEA in either cell culture. mRPL13a and GAPDH were used as housekeeping genes. Results are expressed as mean ± s.d. *n* = 3 biological samples. * indicates significant differences compared to non-treated data (*: *p* < 0.05).

The physiologically relevant concentration of ZEA in brain tissue is predicted to be in the 1–10 nM range in rodents when feeding the animals with non-observed effect-level (NOEL) doses [46]. Former studies have also shown that ZEA is bioactive even at a low, 10 nM concentration in several cell-based systems [40,47,48,49]. This observation was confirmed via our preliminary screening, so detailed analyses were performed using only 10 nM of ZEA. According to our data, 48 h treatment with ZEA at 10 nM significantly increased the viability of each culture type (Figure 4A).

ZEA is known to be capable of binding to both the ERα and ERβ nuclear estrogen receptors of mammals, and already induces estrogenic responses at 1–10 nM concentrations [50,51], with higher Kd than estrogen (17β-estradiol, E2; [52]). Thus, dietary uptake of mycoestrogens may disrupt the natural signaling pathways of E2 [53]. Based on these former observations, the effect of ZEA on the expression of genes encoding nuclear estrogen receptors was also investigated in all three types of cultures. First, we examined the transcription rates of the ERα and ERβ genes, comparing them to the expression of housekeeping genes (mRPL13a and GAPDH). Analysis of the amplification curves (ΔCq) revealed that both types of estrogen receptors were expressed in both neurons and astroglia cells, but that their amount differed between culture types (Figure 4B). Astroglial cultures expressed similar amounts of ERα and ERβ receptors (Figure 4B). In cultures containing neurons, on the other hand, higher expressions of ERα and lower levels of ERβ were observed compared to astrocytes (the amplicon lengths were nearly the same, see Table A1).

To evaluate the effects of ZEA administration, we quantified the expression of ER mRNAs upon 48 h treatment with 10 nM of ZEA, normalized to the corresponding control ΔCq values. According to our data, the expression of ERα was decreased in pure and mixed neuronal cultures and increased in astroglial cultures (Figure 4C). ERβ expression, on the other hand, was not altered via ZEA treatment in either culture type (Figure 4D). As changes in the expression of ERs are generally linked to E2 pathway induction [54], the observed increase in cell viability after ZEA administration suggests that there are certain metabolic changes in action.

### 2.4. ZEA Does Not Affect the Number of Mitochondria in Astrocytes

**Figure 5:**
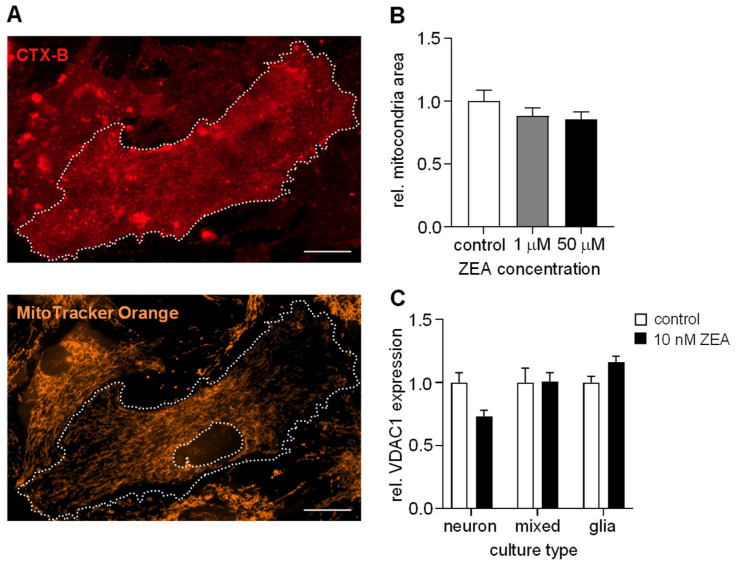
**ZEA does not influence the number of mitochondria, but reduces the expression of VDAC1, an ion channel of the outer mitochondrial membrane.** (**A**) Cholera Toxin B-Alexa647 was used to visualize the cell membrane (white, dotted line), while mitochondria and nuclei were stained using MitoTracker Orange7510 and DAPI, respectively. The relative area of mitochondria was determined as the ratio of mitochondria/cytoplasm areas, using ImageJ 1.54p software. Scale bars, 20 μm. (**B**) Effects of ZEA on the relative area of mitochondria in astroglial cultures. Results are expressed as mean ± s.e.m. (*n* = 12–15). (**C**) VDAC1 relative expression in ZEA-treated cells (black) compared to the control, non-treated cultures (white), analyzed via qRT-PCR. mRPL13a and GAPDH were used as housekeeping genes. Results are expressed as mean ± s.d. *n* = 3 biological samples.

Estrogens are well known to influence mitochondrial activity under both normal physiological and pathological conditions, such as by controlling the transcriptional regulation of mitochondrial proteins, interacting with mitochondrial ER receptors, and stabilizing the activity and structural morphological integrity of the mitochondrial respiratory electron transport chain [55]. To decide whether the observed ZEA-mediated increase in cell viability is due to a change in mitochondrial function or volume, the extent of the mitochondrial network was estimated in the presence or in the absence of ZEA in astroglia cells (Figure 5A). To do so, the mitochondrial network was stained with MitoTracker Orange, and its area was normalized to the area of the cytoplasm. Our analyses did not reveal any significant differences in the mitochondrial area compared to the control cultures (Figure 5B).

For further analysis, we also compared the expression of the mitochondrial marker voltage-dependent anionic channel (VDAC1) via qRT-PCR. The relative expression of VDAC1 was compared to the expression of housekeeping genes (mRPL13a and GAPDH). As shown in Figure 5C, ZEA treatment did not substantially alter the expression of the VDAC1 gene. No changes were observed in mixed and astroglial cultures; however, in neuronal cultures, the expression of the VDAC1 gene was slightly but not significantly reduced.

Taken together, neither immunocytochemical evaluation nor mitochondrial marker gene expression assays were able to detect significant changes in the mitochondrial network in any of the investigated culture types after ZEA treatment. Thus, our results suggest that increased cell viability may be primarily due to other metabolic causes.

### 2.5. ZEA Alters Spontaneous Neuronal Activity in Mixed-Cell Cultures

**Figure 6:**
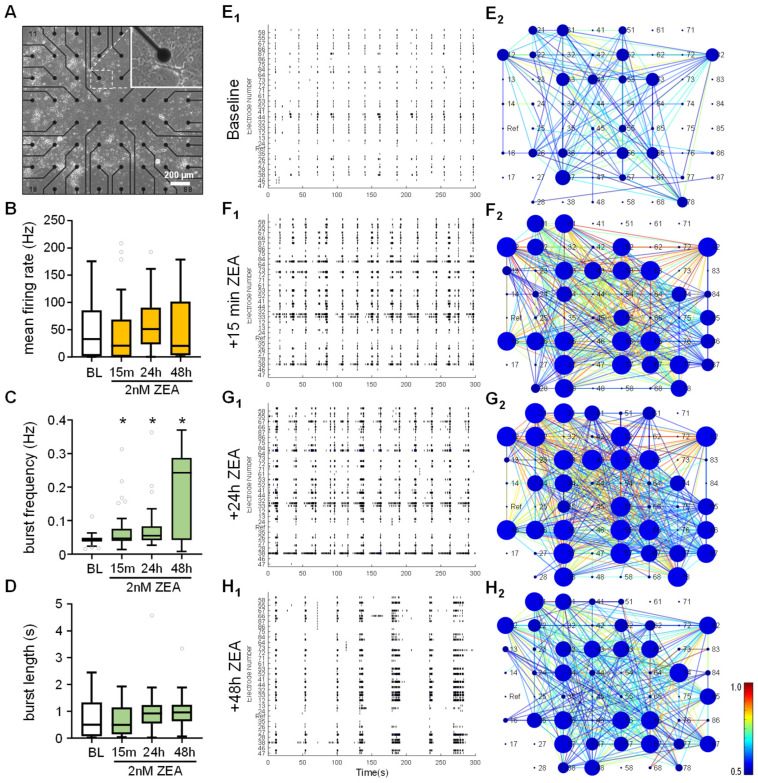
**ZEA rapidly alters neuronal activity by affecting firing patterns.** (**A**) Overview of in vitro developed neuronal network, cultured on the surface of an MEA chip. (**B**–**D**) Electrophysiological parameters measured via multi-electrode array before and after the addition of 2 nM of ZEA to the culture medium. Results are represented as box plots (interquartile range withmedian and outliers). *: *p* < 0.05, *n* = 16–40. (**E_1_**–**H_1_**) Representative spike trains obtained from MEA60 chips depicted as raster plots from 5 min-long recordings before (**E**) and after (**F**: 15 min, **G**: 24 h, and **H**: 48 h) 2 nM ZEA treatment. (**E_2_**–**H_2_**) Cross-correlograms of the respective recorded network activity, where the node size represents the total number of connections for the given node (electrode) and edge color shows the relative coincidence value between the interconnected nodes.

It is also known that estrogen can alter the firing activity of neurons [56]. To investigate whether the mycoestrogen ZEA has a similar effect on neuronal cultures, multi-electrode array (MEA) electrophysiology was performed to investigate the spontaneous electrical activity of neuronal cultures over time [57]. Dissociated neurons were cultivated on top of the electrode surfaces of MEA chips (Figure 6A). Following a baseline recording, 2 nM of ZEA was added to the cultures and spontaneous neuronal activity was monitored in a non-invasive way after 15 min and 24 or 48 h later, based on changes in local field potentials (LFPs; Figure 6B–F).

Primary neurons grown on MEA chips formed dense neural networks (Figure 6A) and exhibited repetitive and robust bursting activity (Figure 6E_1_), characterized by a high ratio of spikes appearing within the bursts (74.3 ± 5.0%, mean ± s.e.m, *n* = 48). ZEA treatment did not change the firing activity, so the mean frequency (Figure 6B) and the burst length parameters (Figure 6D) were unaffected upon ZEA treatment. The ratio of active electrodes remained similar throughout the recordings (baseline: 49.95%; +15 min ZEA: 41.45%; +24 h ZEA: 38.1%; +48 h ZEA: 31.3% and Figure 6E_2_–H_2_, where the blue circles over the given electrodes indicate the nodes of the cross-correlograms, with their diameter representing the total number of active connections within the network). The activity patterns were also characterized by the network level, with the firing coincidence calculated among the electrodes of the MEA60 chip (Figure 6E_2_–H_2_). Cross-correlation maps clearly showed that simultaneous firing became dominant after ZEA administration. Importantly, the pattern of spontaneous neuronal activity significantly changed as the burst frequency values significantly increased (Figure 6C). These effects were observed within 15 min of adding ZEA to the culture medium (Figure 6C,F_1_,F_2_), which was further enhanced by the longer survival. Importantly, small bursts concatenated into longer “superbursts”, which were predominant after 48h treatment (Figure 6H_1_). The ratio of spikes within bursts also slightly increased (81.0 ± 6.8%, mean ± s.e.m, *n* = 19), but this change was not significant. These data show that ZEA rapidly influences the spontaneous electrical activity and synchronization of neuronal networks.

## 3. Discussion

Mycotoxins produced by *Fusarium* spp. molds are worldwide pollutants of food and feed [1]. While most of them are known to have a wide range of biological effects, their neurotoxic potential and the underlying mechanisms of their toxicity are still not clear. The literature is contradictory regarding their toxic effects in the brain, primarily due to the diversity of in vivo and in vitro models used. In addition, certain species have different tolerance to these toxins [5,29]. In this study, we aimed to compare the effects of fumonisin B1, deoxynivalenol, and zearalenone on mouse primary cultures containing neurons and astrocytes in different amounts to characterize the primary cellular targets of the different mycotoxins. Moreover, in the case of ZEA, we also performed detailed analyses to clarify its mode of action in our in vitro system.

### 3.1. Effects of Fumonisin B1 on Neural Cultures

Previously, several studies have shown that FB1 has neuronal effects, as it evokes neurotoxicity, caused by oxidative stress or apoptosis [22] and alterations in myelin formation [20]. In contrast, Bódi et al. recently showed only a mild increase in neuronal intrinsic excitability in vitro, as only a high concentration (100 μM) of FB1 evoked a significant increase in the electrophysiological properties of either hippocampal cell cultures or brain slices. Moreover, changes in neuronal activity and excitability under in vivo conditions were not detected [58]. In addition, FB1 did not cause cell death in astroglia cultures within the 1 nM–50 μM concentration range used in our experiments, but slightly and significantly increased cell viability. This observation is in line with previous studies. For example, Kwon et al. showed that FB1 (0.5–75 µM) was not cytotoxic after 5 or 10 days of exposure in primary rat astro- and oligodendroglial cultures [59]. Similar results were obtained from differentiated primary rat astrocyte cells [60] or in a glioblastoma cell line [61]. On the other hand, incubation with 50 µM of FB1 for 8 days caused cell death in primary mouse astrocyte cultures [62].

In the case of neuronal and mixed cultures, FB1 also caused a small (<20%) but significant, dose-independent increase in cell viability at low concentrations (<10 µM). This is consistent with the findings of Harel et al., where primary rat hippocampal cultures did not show signs of cell death despite a 48 h application of FB1 at 10–40 µM concentrations [18]. Other reports also showed non-toxic effects on different culture types, even at higher (200 μM) concentrations [20,63,64]. Notably, FB1 was not toxic to human SH-SY5Y neuroblastoma cells at 200 µM after 24 h [64]; however, another study reported toxicity over 100 µM after 48 h [65].

In our pure neuronal cultures, however, 50 µM of FB1 drastically reduced cell viability. This effect was independent of the duration of treatment (24 vs. 48 h) and was not observed when astroglial cells were also present (mixed culture). In a recent study, Domijan et al. reported that besides modifying membranes and the induction of oxidative stress, FB1 can inhibit the mitochondrial transport chain I complex and deregulate calcium signaling [64]. They observed that FB1 administration (0.5–200 μM) significantly increases the level of glutathione (GSH) in astrocytes. As GSH is the major antioxidant in the CNS and astrocytes are the major source of GSH in the brain [66], the excreted GSH by astrocytes can reduce the oxidative stress in neurons [67]. Since FB1 is able to alter neuronal excitability [58] and increase glutamate-induced Ca^++^-response [58], it is feasible that the neuroprotective effect of glial cells is exerted via the regulation of glutamate levels [68]. In addition, other mechanisms such as neurotrophic factor release, the recycling of ascorbate to directly scavenge ROS, or even the release of glial exosomes are also quite conceivable [69,70].

Taken together, these results indicate that fumonisin B1 at higher concentrations is more cytotoxic to neurons, and that the presence of astrocytes can partially compensate for FB1-mediated neuronal toxicity.

### 3.2. Effects of Deoxynivalenol on Neural Cultures

Deoxynivalenol is one of the most abundant *Fusarium* mycotoxins. In the last two decades, several studies have indicated DON contamination in harvested cereals and animal feeds [71,72] and revealed exposures above the tolerable daily intake (TDI: 1 μg/bw kg) to DON in the German and Austrian populations [73,74], where the provisional daily intake (PDI) exceeded the TDI in 12% and 33% of the volunteers, respectively. As some effects triggered by DON can occur in vivo in the brain at concentrations close to the TDI [24], neurological damage is a valid risk. Former studies have shown that DON can reach neuronal tissues [23,75], and in concentrations exceeding 10 μM, it can reduce the integrity of the blood–brain barrier [76]. It is also known that DON decreases the brain protein levels and the activity of the antioxidant system and induces oxidative stress, apoptosis [33], and can cause neuroinflammation [30]. DON has also been reported to have neuroendocrine effects [29].

Based on our results obtained from primary neural cultures, DON acts in a cell type-specific manner. Cell viability was reduced in all three culture types in a concentration- and exposure time-dependent manner; however, astroglial cells were found to be the most sensitive to DON (cell viability was decreased above 0.2 μM concentrations in glial and above 1 μM doses in mixed and neuronal cultures). The sensitivity of glial cells is consistent with previous work, where DON was found to be cytotoxic in rat glia cell cultures in vitro within the 10–100 µM range [77]. In this study, DON also reduced the viability of astroglial cells: the IC50 dose calculated for the glial cells (31 μM) is comparable with our finding (24 μM). However, in this work, microglial cells were primarily the most sensitive population. Similar effects on the human astroglial cell line, STTG-1, were also demonstrated, but only at higher doses (>25 μM). In addition, DON has also been shown to inhibit the glutamate uptake of astrocytes, which is a key astroglial function [77].

The effect of DON on the viability of rodent neurons is, to our best knowledge, a new, unpublished scientific result. Recently, Wang et al. presented neuronal data on DON toxicity using piglet hippocampal cells and PC12 cell lines [78,79]. The piglet neurons were already sensitive to DON above 400 nM, which can be explained by the fact that swine are more susceptible to DON than mice or rats [29]. Apoptosis and neuroinflammation are the most feasible mechanisms behind the neurotoxicity of DON [77,78]. According to our data, DON also acts in a cell type-specific manner, and it is likely that the actual tissue concentration of DON determines its glial and/or neuronal effects.

### 3.3. Effects of Zearalenone on Neural Cultures

Little is known about the direct effects of short or continuous exposure to ZEA mycoestrogen on brain function and development. Former publications have shown that besides oxidative and DNA damage, apoptosis, or necrosis caused by ZEA, its main prominent action is mediated through the estrogenic signalization pathways [53]. Both types of nuclear estrogen receptors, ERα and ERβ, are expressed and play a vital role in neuronal development, and their expression is dynamically regulated during critical periods of cortical development [80]. Under physiological conditions, E2 is able to bind to the α-receptor with greater affinity [81], and E2-induced changes in the expression of estrogen-sensitive genes ultimately lead to the regulation of central nervous system development and neuronal differentiation and affect cell migration, viability and cell death, and synaptic plasticity [82].

In this study, expressions of both estrogen receptor mRNAs (ERα and ERβ) were detectable via RT-qPCR in all culture types. The expression of ERβ receptors was lower than ERα receptor levels in cultures containing neurons. There are limited data supporting the roles of ERα signaling in astroglial cells, including effects on morphology, proliferation, and reactive astrogliosis (reviewed in [83]). The astrocyte-specific knockout of aromatase results in impaired reactive astrogliosis and decreased activation of the JAK–STAT3 pathway after brain injury [84]. On the other hand, the reduction in the ERα level in neurons may be due to developmental immaturity, possibly protecting the brain from hormonal interference during early development. Similar observations are reported in the literature [85,86,87,88], with the note that the pattern of the mRNA expression and protein expression of these receptors is generally correlated [85]. In the adult brain, E2 has been shown to mostly reduce the expression of ERα mRNA [54]. Moreover, 10 nM of ZEA exhibited similar effects in neuronal cultures after 48 h of exposure. In contrast, in astroglial cultures, ZEA caused a significant increase in ERα expression. This is consistent with Frago et al., who reported that E2 increased the amount of ERα mRNA in astroglial cultures obtained from female rats [89]. Although in the study, E2 reduced the expression of ERβ in astroglial cells, as well, we did not detect a similar effect of ZEA treatment in mouse astroglial cultures.

In mixed cultures, no significant change in ER–receptor–mRNA levels was detected after ZEA treatment. Since the sex of the embryos and newborn pups used to establish the cell cultures was not determined due to technical limitations, further investigations are needed to investigate if ZEA exerts sex-dependent effects on neurons, similarly to E2. It should be noted that in other systems, e.g., using neuroblastoma cell lines or cerebellar granule cells, opposite effects were exhibited upon ZEA administration [42,90], further strengthening the cell type-specific action of ZEA.

Some studies investigated the direct toxic effects of ZEA on neural cell types [34,36,43,53]. In our in vitro assay, ZEA increased cell viability in all cell culture types, even at 10 nM. Theoretically, higher cell viability can be achieved via increased proliferation and/or metabolism. Neurons are non-dividing cells; thus, the potential proliferating effect can be excluded. It is also known that estrogens control the transcriptional regulation of mitochondrial proteins and influence mitochondrial activity under both physiological and pathological conditions via ERβ receptors by, e.g., affecting the activity of the mitochondrial respiratory electron transport chain, reducing ROS production, balancing the expression of fusion and fission proteins, or changing the structure of the mitochondrial network [55]. According to our results, ZEA-dependent changes in the extent of the mitochondrial network (determined by the relative area or VDAC1 expression) were not detected. Alternatively, exposure to ZEA can interfere with the oxidoreductive metabolism of mitochondria, and may affect the reductive capacity of the cells by increasing the SOD and GSH levels [36,40,91]. On the other hand, in neuroblastoma cell lines, ZEA induced oxidative stress and reduced cell viability [34] only in 1000× higher, presumably non-physiological concentrations [46,92].

Importantly, the effects of ZEA on neuronal activity and network firing patterns are a new finding. The generally observed burst firing in our cultures is driven by excitatory network episodes due to the dense glutamatergic connections among the cells [93]. ZEA has a strong potentiating effect by increasing the frequency of bursts, but it does not influence the overall firing rate. Besides the individual cell activities, ZEA rapidly promotes spontaneous network burst activity and network synchronization, even after 15 min administration. It also induces the formation of highly synchronized “superbursts”. This phenomenon resembles the effects of estrogen on neuronal cultures, where burst activity has been shown to be more apparent in the presence of estrogen [94].

## 4. Conclusions

Our results strengthen the cell type-specific effects of *Fusarium* toxins on the nervous system. The data obtained from cell cultures with different neuronal and/or astroglial compositions support the importance of identifying the cellular targets of in vivo mycotoxin exposure.

## 5. Materials and Methods

### 5.1. Animal Handling

CD1 wild-type mice were purchased from Charles River Laboratories (Wilmington, MA, USA; organism: RRID:IMSR_CRL:22). Mice were housed at 22 ± 1 °C, with 12 h light/dark cycles and ad libitum access to food and water. Experiments were carried out in accordance with the Hungarian Act of Animal Care and Experimentation (1998, XXVIII) and with the directive 2010/63/EU of the European Parliament and of the Council of 22 September 2010 on the protection of animals used for scientific purposes [95]. Experimental protocols were approved by the Animal Care and Use Committee of Eötvös Loránd University and the National Food Chain Safety Office. Sacrificing the animals to obtain brain tissue and prepare primary neuronal cultures is regarded as organ donation. All possible efforts were made to minimize the number of animals used.

### 5.2. Drug Preparation

Fumonisin B1 (FB1, Tocris Bioscience, UK), deoxynivalenol (DON, Tocris Bioscience, UK), and zearalenone (ZEA, Tocris Bioscience, UK) were dissolved in 10% DMSO (FB1), 10% ethanol (DON), or in 10% ethanol–50% DMSO (ZEA), respectively. Cells were treated with mycotoxins in a 1 nM to 50 μM concentration range, for 24 or 48 h. Control wells were treated with the correspondent solvent.

### 5.3. Preparation of Cell Cultures

The experiments were performed on mixed (where both neurons and astroglia cells were present) and on pure neuronal or astroglial primary cultures. Primary cultures of embryonic cortical neurons were prepared from pregnant CD1 mice on embryonic days 14–15, according to Tárnok et al.,2008 [96]. As sex-selective grouping of embryos from that age is technically challenging [97,98] and would expand the cell isolation time enormously, the sex of the embryos/pups used for the preparation of the cell cultures was not determined. Briefly, under aseptic conditions, cortices were isolated, cleaned from meninges, and incubated in 0.05% trypsin-EDTA solution (Gibco, Thermo Scientific, Hungary) for 15 min at 37 °C. After a brief centrifugation step, cells were triturated in NeuroBasal media (Gibco, Thermo Scientific) supplemented with 2% B27 (Gibco, Thermo Scientific, Hungary), 5% FCS (PAN-Biotech, Germany), 0.5 mM of Glutamax (Gibco, Thermo Scientific, Hungary), 40 μg/mL of gentamycin (Hungaropharma Ltd., Hungary), and 2.5 μg/mL of amphotericin B (Sigma-Aldrich-Merck, Hungary), and filtered through a sterile polyester mesh with a 42 μm pore size (EmTek Ltd., Hungary). Cells were seeded onto poly-L-lysine (PLL; Sigma-Aldrich-Merck, Hungary)-coated 96-well plates (Greiner Bio-One, Hungary) at 8 × 10^4^ cells/well density. For microscopy, 1.5 × 10^5^ cortical neurons were seeded onto poly-L-lysine–laminin (1 μg/cm^2^ Sigma-Aldrich-Merck, Hungary)-coated glass coverslips in 24-well plates. Cells were cultivated at 37 °C in a 5% CO_2_/95% air atmosphere. Neurons were cultured in Neurobasal medium (Thermo Scientific, Hungary), supplemented with 2% B27 (Thermo Scientific, Hungary), 0.5 mM of Glutamax (Gibco, Thermo Scientific, Hungary), 40 μg/mL of gentamycin (Hungaropharma Ltd., Hungary), and 2.5 μg/mL of amphotericin B (Sigma-Aldrich-Merck, Hungary). Then, 5% FCS (PAN-Biotech, Germany) was included in the culture medium until DIV1 (pure neuronal cultures) or DIV4 (mixed neuronal–glial cultures). Complete and partial (1:2) medium changes were performed on DIV1 and DIV4, respectively. Pure neuronal cultures were treated with cytosin–arabinofuranoside (CAR, 10 μM; Sigma-Aldrich-Merck, Hungary) 24 h after plating to prevent the further division of non-neuronal cells. In the case of mixed cultures, cells were treated with 10 μM CAR only on the 6th day after plating. Mycotoxin treatments were performed on the 7–8th day of cultivation (see further details in the text).

The primary cultures of astrocytes were prepared from 1 to 4 day-old CD1 mice, according to Tárnok et al., 2010 [99]. Briefly, cerebral cortices were isolated, freed from meninges, and incubated in trypsin-EDTA [0.5 mg/mL in phosphate-buffered saline (PBS) pH = 7.4, Gibco, Thermo Scientific, Hungary] and 5 mg/mL of DNase solution (Sigma-Aldrich-Merck, Hungary) for 15 min at 37 °C. After brief centrifugation, cells were triturated in high-glucose Dulbecco’s Modified Eagle Medium (HDMEM, Sigma-Aldrich-Merck, Hungary) supplemented with 10% fetal calf serum (FCS, PAN-Biotech, Germany), 40 μg/mL of gentamycin, and 2.5 μg/mL of amphotericin B (all from Sigma-Aldrich-Merck, Hungary), and filtered through a sterile polyester mesh with a 42 μm pore size (EmTek Ltd., Hungary). The cell number was determined via trypan blue exclusion. Cells were seeded onto poly-L-lysine-coated Petri dishes at 3–4 × 10^5^ cells/cm^2^ density in DMEM containing 10% FCS, 2 mM of L-glutamine, 2.5 μg/mL of amphotericin B, and 40 μg/mL of gentamycin. Cells were cultivated at 37 °C in a 5% CO_2_/95% air atmosphere. Astroglial cultures were used for further experiments after reaching confluency (7–8 days in vitro). For the experiments, cells were reseeded into poly-L-lysine-coated 96-well plates or onto 12 mm-diameter coverslips in 24-well plates in a 2.5 × 10^4^ cells/cm^2^ density.

### 5.4. Cell Viability Assay

The viability of the cultures was measured 24 h or 48 h after mycotoxin treatment via MTT assay, as described by Tárnok et al., 2008 [96]. Briefly, cells grown in 96-well plates were treated with 3-(4,5-dimethylthiazol-2-yl)-2,5-diphenyltetrazolium bromide (MTT, Sigma-Aldrich-Merck, Hungary) at a final concentration of 200 μg/mL. After 35–40 min incubation, cells and formazan crystals were dissolved in acidic (0.08 M HCl) isopropanol (Sigma-Aldrich-Merck, Hungary). Optical density was determined at a measuring wavelength of 570 nm against 630 nm as a reference with a Multiskan EX ELISA reader (Thermo Scientific, Hungary). Assays were carried out on at least 6 parallel wells of 3 independent cultures. Viability data were determined as averages and standard errors of the mean and were expressed as a percentage of the control cultures. Data were compared using Student’s *t*-test (*p* < 0.05).

### 5.5. Immunocytochemistry and Microscopic Analysis

Cells grown on 12 mm-diameter coverslips in 24-well plates were fixed with 4% paraformaldehyde (TAAB Laboratories Equipment Ltd., UK; *w*/*v* in PBS), permeabilized with 0.1% Triton-X-100 in phosphate-buffered saline, and blocked in 2% bovine serum albumin in phosphate-buffered saline.

To detect the different cell types in neural cultures, cells were immunostained using anti-IIIβ-tubulin (mouse, 1:1000; EXBIO, Czech Republic), anti-GFAP (chicken, 1:1000, Synaptic Systems, Germany), and anti-Iba1 (goat, 1:500, Novusbio, Bio-Techne, Hungary) antibodies overnight at 4 °C. Anti-mouse Alexa Fluor-DyLight594 (Vector Laboratories, UK), anti-chicken Alexa Fluor-647 (Invitrogen, Thermo Scientific, Hungary), and anti-goat Alexa Fluor-488 (Invitrogen, Thermo Scientific, Hungary) were applied in a 1:500 dilution for 1h at RT. Preparations were mounted with bis-benzimide (Sigma, Hungary)-containing Mowiol 4.88 (for nuclei labeling, Polysciences, Germany).

To stain the mitochondria of astroglia cells, 10 nM of MitoTracker Orange (Thermo Scientific, Hungary) was applied at 37 °C for 25 min before fixing (in the presence or absence of ZEA). After the fixation, cell membranes were labeled using Cholera Toxin B-Alexa647 (50 μg/mL, Invitrogen, Thermo Scientific, Hungary) for 30 min at RT. Preparations were mounted with bis-benzimide-containing Mowiol 4.88 (for nuclei labeling, Polysciences, Germany).

Microscopic images were acquired with an inverted AxioObserver Z1 wide-field fluorescent microscope (Zeiss, Hungary) with a LD Plan Neofluar 20×/0.4 NA (Zeiss, Hungary) or an α-PlanApoChromat 63×/1.46 NA oil immersion objective (Zeiss, Hungary) using Colibri-LED illumination and the appropriate filter sets. Images from sister cultures for the same experiment were recorded with identical microscope settings and analyzed in the same way.

Mitochondrial area was determined in projected z-stack images of astrocytes taken at 63× magnification. Cholera Toxin B and DAPI staining was used to delineate the cell shape and nucleus, respectively, using ImageJ/Fiji 1.54p software [100]. MitoTracker Orange-positive objects within the cytoplasm were separated from the background via intensity thresholding, and their total area was determined to calculate the (total mitochondrial area)/(cytoplasmic area) ratio.

### 5.6. Quantitative Real-Time PCR (qRT-PCR)

Cultures were lysed and RNA samples were obtained using Quick-RNA MiniPrep (ZYMO Research, Hungary). Reverse transcription was performed with the Maxima First Strand cDNA Synthesis for the RT-qPCR kit (Thermo Scientific, Hungary), according to the manufacturer’s instruction. Messenger RNA expression was investigated using the Maxima SYBR qPCR Master Mix (Thermo Scientific, Hungary) with specific primers (see Table A1, in the Appendix A). The qPCR run was performed using a CFX96 (C1000 Touch) from Bio-Rad Laboratories (USA) with the following settings: 1 cycle at 95 °C for 10min, 40 cycles at 95 °C for 15 s followed by 55 °C for 30 s, and 72 °C for 30 s. Cq and RFU values were obtained from Bio-Rad CFX Manager v3.0 software (Bio-Rad, USA). The relative expression of the interested genes was calculated using the ΔΔCq method and triplicated samples.

### 5.7. MEA Recordings and Analysis

In MEA experiments, cells were seeded onto MEA60-4Well-PT (Qwane Biosciences, Switzerland), 60ThinMEA 100/10 ITO (Multichannel Systems, Germany), or 60MEA 200/30 ITO (Multichannel Systems, Germany) multi-electrode chambers at 4 × 10^4^ (MEA60-4Well-PT) or 4–5 × 10^5^ (60ThinMEA 100/10 ITO and 60MEA 200/30 ITO) cells/well densities. Prior to seeding the cells, the surface of the multi-electrode arrays was coated with poly-L-lysine (2 µg/cm^2^, Sigma-Aldrich-Merck, Hungary) and laminin (4.2 µg/cm^2^, Sigma-Aldrich-Merck, Hungary).

Spontaneous extracellular field potentials were recorded at 37 °C (Temperature Controller TC01102, Multichannel Systems, Germany) using a USB-1060 INV Microelectrode array System (Multichannel Systems, Germany) with a MEA Amplifier with a Blanking Circuit (Multichannel Systems, Germany) amplifier at a sampling rate of 20 kHz/channel. Signals were high-pass filtered at 300 Hz and low-pass filtered at 3000 Hz with a Butterworth 2nd-order filter. Spontaneous firing was recorded for 10 min before the addition of the toxins (baseline) and repeated after the toxin treatment for another 10 min. Recorded spikes were detected via a threshold crossing method (Thr.%: −1.1) and were sorted via the k means algorithm. The Thr.% shows the threshold value in A/D counts and the associated voltage that was used to collect the waveforms. The spike timestamps were extracted using the Plexon Offline Sorter (Plexon Inc., TX, USA). Analysis of the spike timestamps, including characterizing spontaneous firing and burst activity, was performed by the NeuroExpress software (https://www.researchgate.net/publication/338623194_NeuroExpress_program_for_analyzing_patch-clamp_data, accessed on 10 June 2025). Network activity graphs were created using MEAnalyzer [101] software, with a 0.5 cross-correlation threshold.

### 5.8. Statistics

For the statistical evaluation of pairs of data sets, Student’s *t*-tests (paired or independent, in case of normal distribution of data) or non-parametric Mann–Whitney tests (in case of non-normal distribution of data) were used. Statistics were calculated using SPSS Statistics v29 (IBM, USA). A *p*-value equal to or smaller than 0.05 was considered statistically significant. The number of independent experiments (*n*) is indicated in the figure legends.

## Data Availability

The data presented in this study are available in this article.

## Abbreviations

The following abbreviations are used in this manuscript:

FB1	Fumonisin B1
DON	Deoxynivalenol
ZEA	Zearalenon
ER	Estrogen receptor
MEA	Multi-electrode array

## Appendix A

**Table A1:** Primer sets used in qRT-PCR analysis. The designed primer pairs recognize all transcriptional variants.

Gene of Interest	Primer	Sequence	Product Length (bp)
Mus musculus estrogen receptor 1 (alpha) (Esr1) (NM_007956.5)	Forward	ATTATGGGGTCTGGTCCTGC	187
	Reverse	TCTTTCCGTATGCCGCCTTT	
Mus musculus estrogen receptor 2 (beta) (Esr2) (NM_207707.1)	Forward	CATTACGGTGTCTGGTCCTGT	189
	Reverse	TTCTCTCCTGGATCCACACTTG	
Mus musculus ribosomal protein L13A (Rpl13a) (NM_207707.1)	Forward	AGGGGCAGGTTCTGGTATTG	191
	Reverse	GGGGTTGGTATTCATCCGCT	
Mus musculus voltage-dependent anion channel 1 (Vdac1)(NM_001362693.1)	Forward	GGCTACGGCTTTGGCTTAAT	199
	Reverse	CAGTGATCTCAGTGCCCAGG	
Mus musculus GAPDH (NM_008084.3)	Forward	TGGTGAAGGTCGGTGTGAAC	106
	Reverse	ATGAAGGGGTCGTTGATGGC	
